# Supplementary figures and images for: Repeatability analysis improves the reliability of behavioral data
Source: PLoS One. 2020 Apr 2;15(4):e0230900. doi: 10.1371/journal.pone.0230900 (PMC7117744; doi:10.1371/journal.pone.0230900)

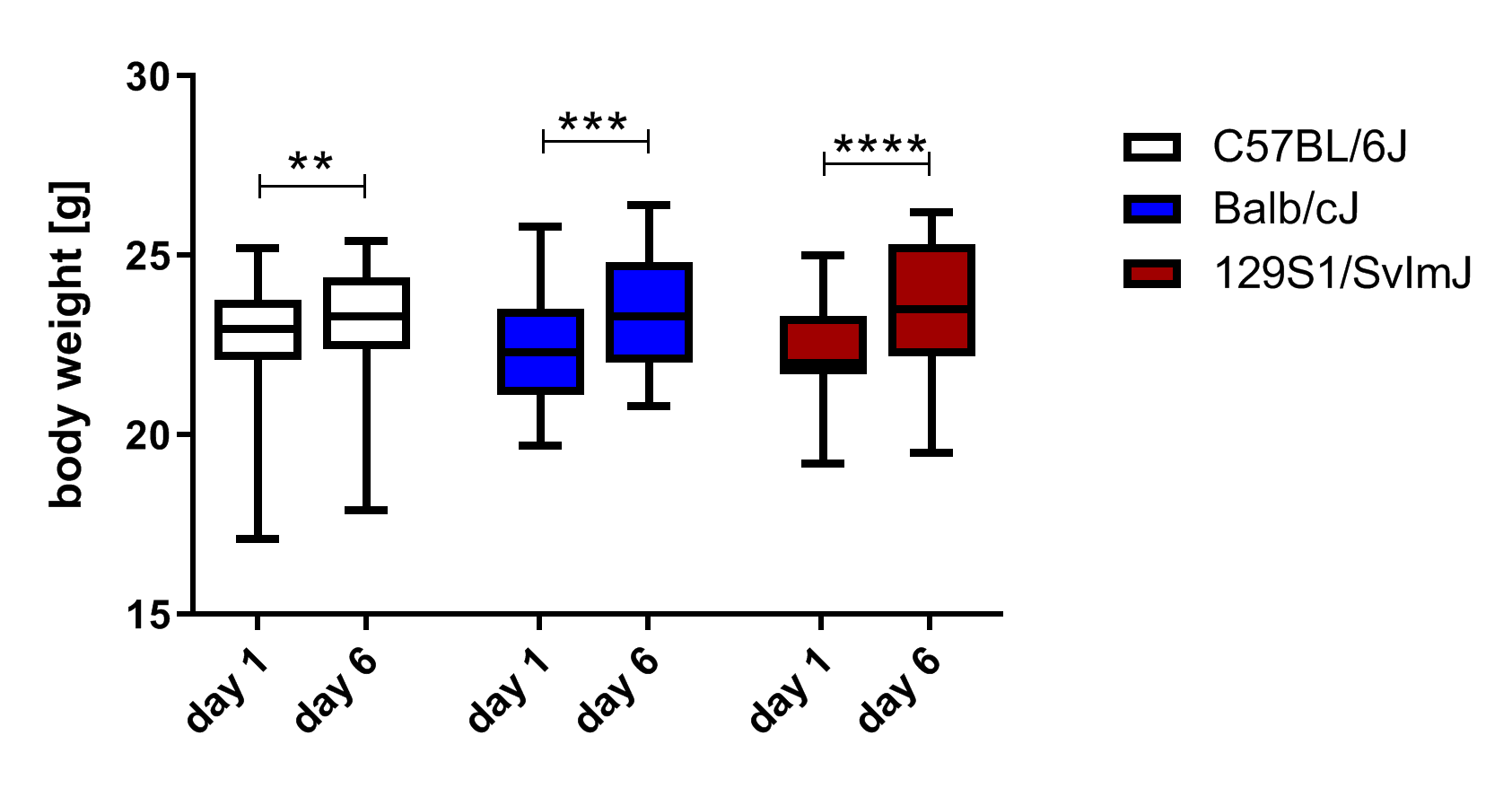

Supplement: S1 Fig — Body weight [g] at day one and six of habituation period per strain were presented as box plot with median and whiskers [2.5, 97.5%] (n = 38 C57BL/6J: p-value = 0.0018, n = 15 BALB/cJ: p-value = 0.0004 and n = 15 129S1/SvImJ: p-value < 0.0001, Wilcoxon matched-pairs signed rank test, two-tailed). (TIF) [file pone.0230900.s001.tif]

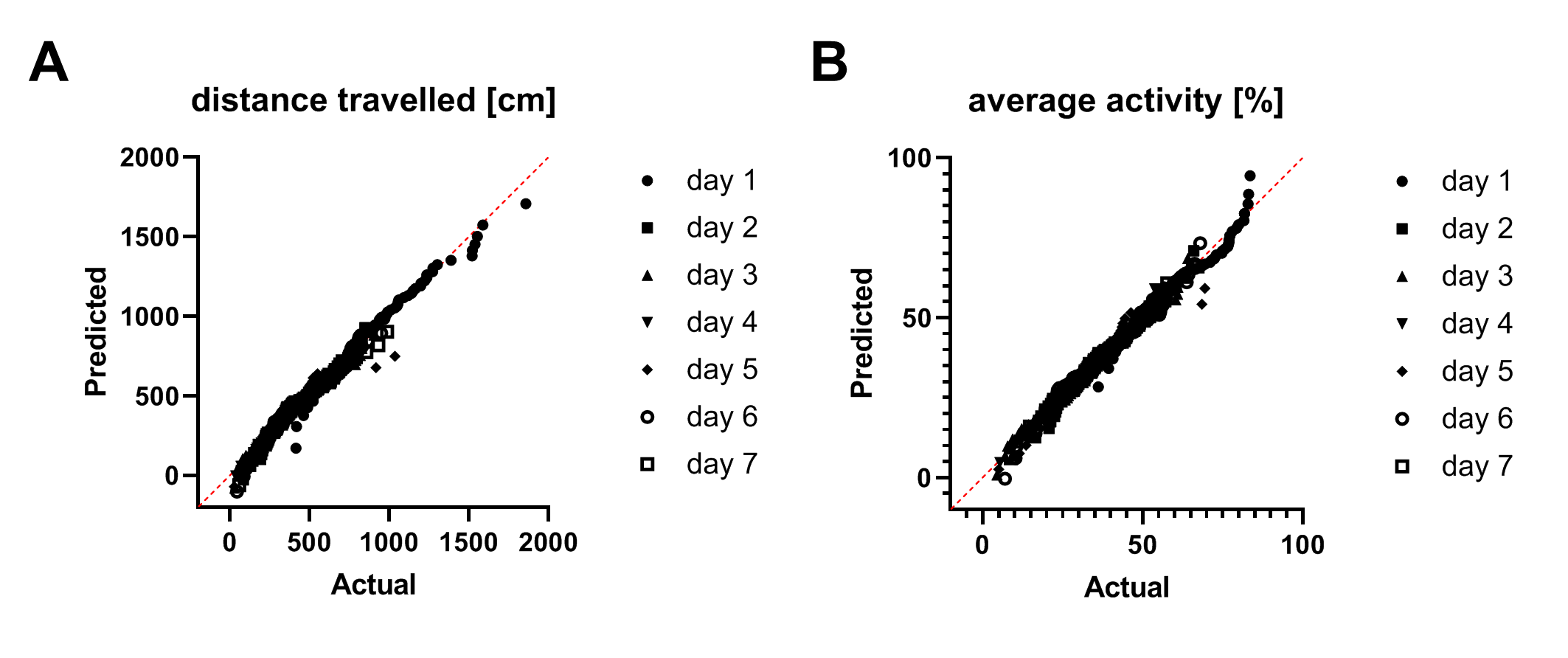

Supplement: S2 Fig — Data sets of distance travelled and average activity were checked for normal distribution using Q-Q-norm plot (n = 38 C57BL/6J, n = 15 BALB/cJ and n = 15 129S1/SvImJ male mice). (TIF) [file pone.0230900.s002.tif]

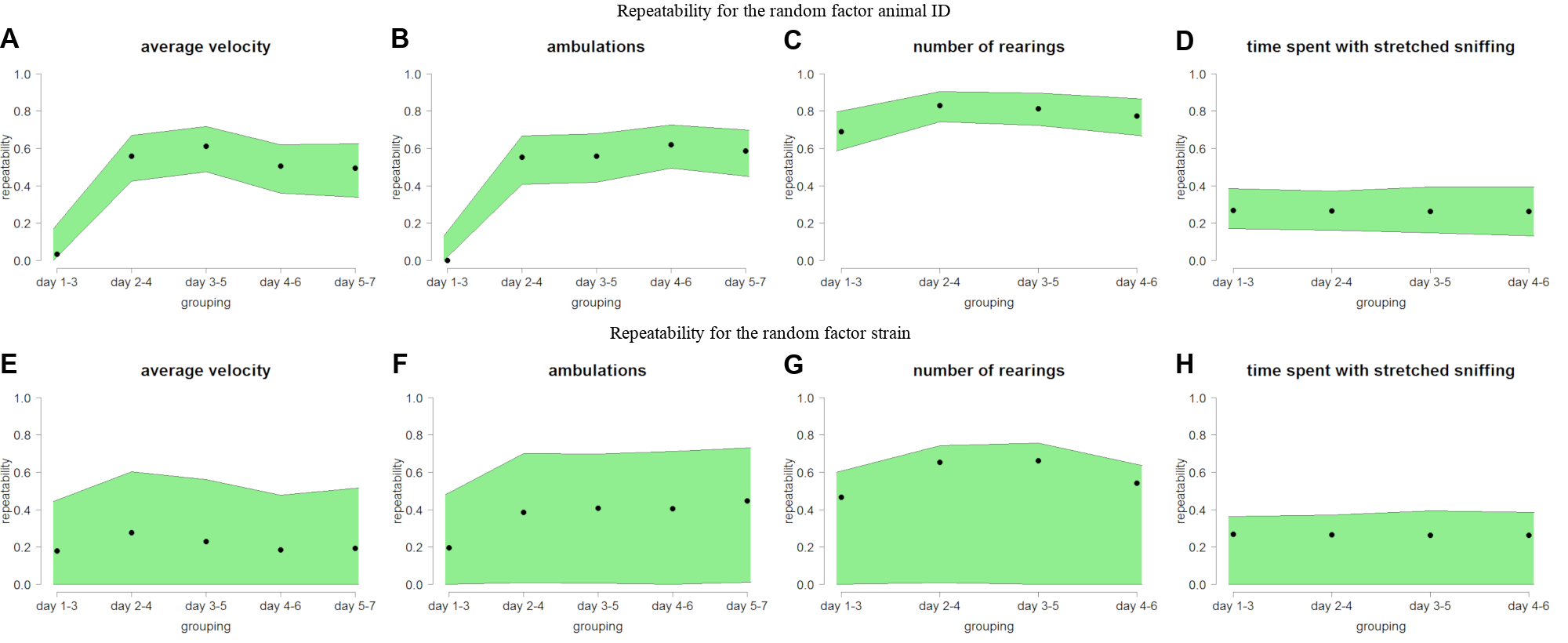

Supplement: S3 Fig — (A—D) Calculated animal ID repeatability value and (E—H) strain repeatability value for the factors (A, E) average velocity, (B, F) number of ambulations, (C, G) number of rearings and (D, H) time spent with stretched sniffing were presented. Each repeatability value (R, black points) was calculated over three adjacent days resulting in five groupings for velocity and ambulations and in four groupings for rearing and sniffing behavior (n = 38 C57BL/6J, n = 15 BALB/cJ and n = 15 129S1/SvImJ male mice). Estimation of repeatability was conducted with a linear mixed-effect model based on Gaussian distribution for velocity and ambulations and with a generalized linear mixed-effect model based on Poisson distribution for rearing and sniffing behavior. The [2.5%, 97.5%] confidence intervals (CI) were displayed in green, resulting from 500 bootstrapping runs and 100 permutations. (TIF) [file pone.0230900.s003.tif]

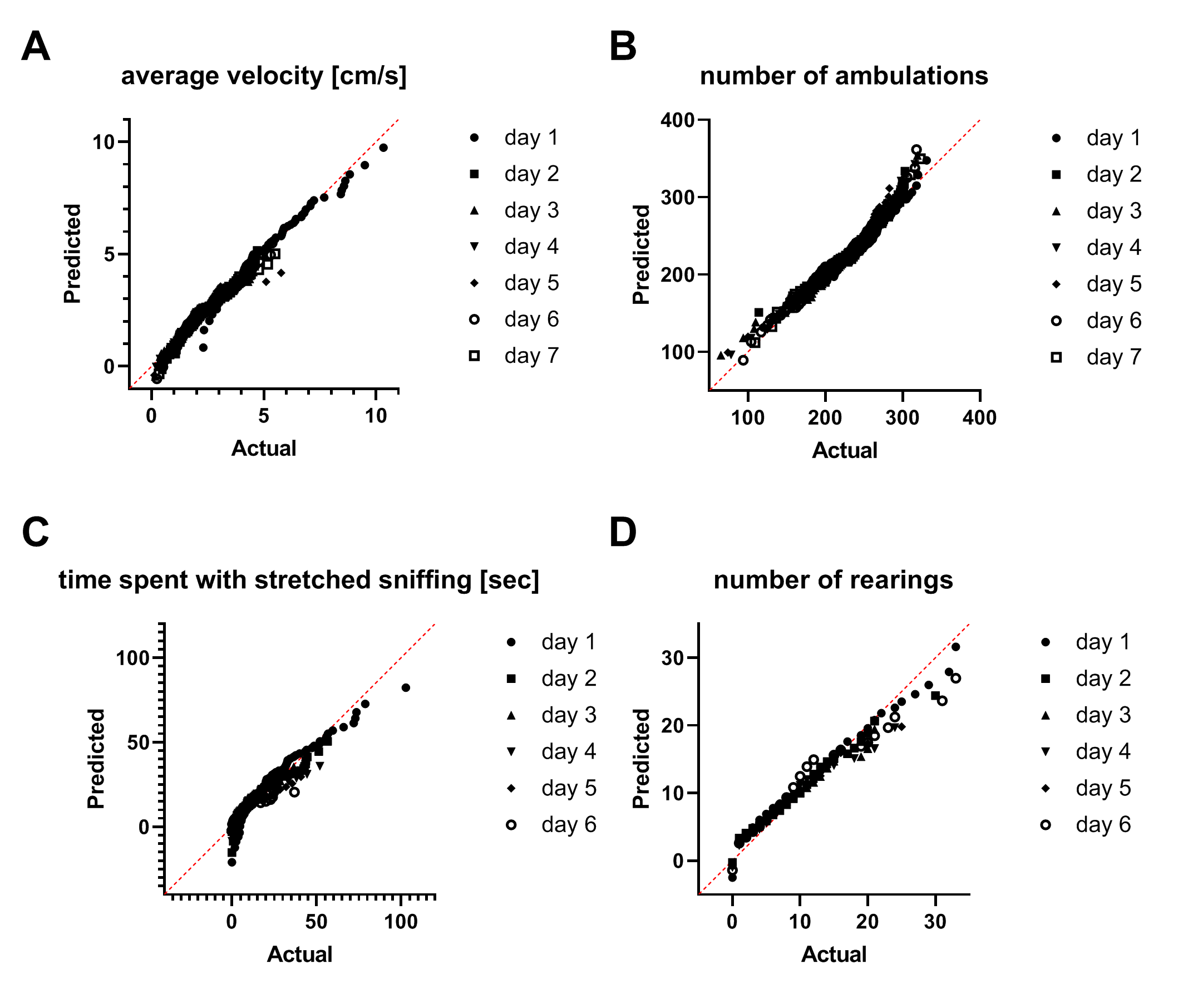

Supplement: S4 Fig — Data sets of average velocity, sum of ambulations, number of rearings and time spent with stretched sniffing were checked for normal distribution using Q-Q-norm plot (n = 38 C57BL/6J, n = 15 BALB/cJ and n = 15 129S1/SvImJ male mice). (TIF) [file pone.0230900.s004.tif]
