# Supplementary material for: Repeatability analysis improves the reliability of behavioral data
Source: PLoS One. 2020 Apr 2;15(4):e0230900. doi: 10.1371/journal.pone.0230900 (PMC7117744; doi:10.1371/journal.pone.0230900)
Supplement: S2 Table — Each repeatability value (R) was calculated over three adjacent days resulting in five groupings. For every factor R, the [2.5%, 97.5%] confidence intervals (CI) and p-values calculated by likelihood ratio test were displayed (n = 38 C57BL/6J, n = 15 BALB/cJ and n = 15 129S1/SvImJ male mice). Estimation of repeatability was conducted with a linear mixed-effect model based on Gaussian distribution. The CI resulted from 500 bootstrapping runs and 100 permutations. (PDF) [file pone.0230900.s006.pdf]

S2 Table. Repeatability values for animal ID as random factor with and without adjustment for the factor strain for distance travelled and average activity for distance travelled and average activity.

	**distance travelled**			**adjusted distance travelled**			**average activity**			**adjusted average activity**		
**grouping**	**R**	**CI**	**p**	**R**	**CI**	**p**	**R**	**CI**	**p**	**R**	**CI**	**p**
**day 1-3**	0.027	[0, 0.166]	0.377	0	[0, 0.15]	0.5	0.199	[0.046, 0.345]	0.00413	0	[0, 0.15]	1
**day 2-4**	0.556	[0.415, 0.675]	9.99E-15	0.445	[0.302, 0.584]	2.66E-09	0.634	[0.501, 0.729]	2.17E-19	0.465	[0.307, 0.605]	4.52E-10
**day 3-5**	0.617	[0.482, 0.726]	2.79E-18	0.544	[0.403, 0.669]	1.3E-13	0.652	[0.528, 0.75]	1.08E-20	0.519	[0.365, 0.649]	2.05E-12
**day 4-6**	0.505	[0.347, 0.62]	3.16E-12	0.445	[0.271, 0.572]	2.52E-09	0.627	[0.506; 0.730]	6.53E-19	0.534	[0.391, 0.657]	4.37E-13
**day 5-7**	0.498	[0.344, 0.626]	6.49E-12	0.438	[0.274, 0.575]	4.58E-09	0.604	[0.465, 0.710]	1.94E-17	0.499	[0.357, 0.626]	1.7E-11

Each repeatability value (R) was calculated over three adjacent days resulting in five groupings. For every factor R, the [2.5 %, 97.5 %] confidence intervals (CI) and p-values calculated by likelihood ratio test were displayed (n = 38 C57BL/6J, n = 15 BALB/cJ and n = 15 129S1/SvImJ male mice). Estimation of repeatability was conducted with a linear mixed-effect model based on Gaussian distribution. The CI resulted from 500 bootstrapping runs and 100 permutations.
