# Supplementary material for: Repeatability analysis improves the reliability of behavioral data
Source: PLoS One. 2020 Apr 2;15(4):e0230900. doi: 10.1371/journal.pone.0230900 (PMC7117744; doi:10.1371/journal.pone.0230900)
Supplement: S3 Table — Each repeatability value (R) was calculated over three adjacent days resulting in five groupings. For every factor R, the [2.5%, 97.5%] confidence intervals (CI) and p-values calculated by likelihood ratio test were displayed (n = 38 C57BL/6J, n = 15 BALB/cJ and n = 15 129S1/SvImJ male mice). Estimation of repeatability was conducted with a linear mixed-effect model based on Gaussian distribution. The CI resulted from 500 bootstrapping runs and 100 permutations. (PDF) [file pone.0230900.s007.pdf]

S3 Table. Repeatability values for strain as random factor for distance travelled and average activity.

	distance travelled			average activity		
**grouping**	**R**	**CI**	**p**	**R**	**CI**	**p**
**day 1-3**	0.177	[0, 0.452]	1.47E-06	0.321	[0.003, 0.651]	4.86E-13
**day 2-4**	0.276	[0, 0.612]	3.57E-10	0.401	[0.005, 0.714]	8.06E-17
**day 3-5**	0.229	[0, 0.51]	3.62E-08	0.363	[0, 0.702]	1.74E-14
**day 4-6**	0.182	[0, 0.491]	1.68E-05	0.297	[0, 0.604]	3.87E-10
**day 5-7**	0.19	[0, 0.459]	2.15E-05	0.307	[0, 0.663]	1.37E-10

Each repeatability value (R) was calculated over three adjacent days resulting in five groupings. For every factor R, the [2.5 %, 97.5 %] confidence intervals (CI) and p-values calculated by likelihood ratio test were displayed (n = 38 C57BL/6J, n = 15 BALB/cJ and n = 15 129S1/SvImJ male mice). Estimation of repeatability was conducted with a linear mixed-effect model based on Gaussian distribution. The CI resulted from 500 bootstrapping runs and 100 permutations.
