# Supplementary material for: Repeatability analysis improves the reliability of behavioral data
Source: PLoS One. 2020 Apr 2;15(4):e0230900. doi: 10.1371/journal.pone.0230900 (PMC7117744; doi:10.1371/journal.pone.0230900)
Supplement: S4 Table — For every factor, the repeatability R, the [2.5%, 97.5%] confidence intervals (CI) and the p-values, calculated by likelihood ratio test, were displayed over the seven-day habituation period for average velocity and number of ambulations and over a six-day habituation period for rearing and sniffing behavior (n = 38 C57BL/6J, n = 15 BALB/cJ and n = 15 129S1/SvImJ male mice). Estimation of repeatability was conducted with a linear mixed-effect model based on Gaussian distribution for average velocity and number of ambulations and with a generalized linear mixed-effect model based on Poisson distribution for rearing and sniffing behavior. The CI resulted from 500 bootstrapping runs and 100 permutations. (PDF) [file pone.0230900.s008.pdf]

S4 Table. Repeatability values with multiple grouping factors: animal ID, strain and experiment for average velocity, ambulations as well as rearing and sniffing behavior.

	repeatability for animal ID			repeatability for strain			repeatability for experiment		
	**R**	**CI**	**p-value**	**R**	**CI**	**p-value**	**R**	**CI**	**p-value**
velocity	0.097	[0.033, 0.179]	2.18E-4	0.098	[0, 0.292]	0.0022	0.014	[0, 098]	0.263
ambulations	0.092	[0.036, 0.169]	5.4E-6	0.277	[0, 0.589]	2.23E-8	0.072	[0, 0.293]	0.0116
rearing	0.047	[0.003, 0.449]	2.33E-20	0.406	[0, 0.503]	2.05E-15	0	[0, 0.034]	1
sniffing	0.032	[0.002, 0.275]	NA	0.032	[0, 0.129]	NA	0.032	[0, 0.112]	NA

NA: close to zero or negative

For every factor, the repeatability R, the [2.5 %, 97.5 %] confidence intervals (CI) and the p-values, calculated by likelihood ratio test, were displayed over the seven-day habituation period for average velocity and number of ambulations and over a six-day habituation period for rearing and sniffing behavior (n = 38 C57BL/6J, n = 15 BALB/cJ and n = 15 129S1/SvImJ male mice). Estimation of repeatability was conducted with a linear mixed-effect model based on Gaussian distribution for average velocity and number of ambulations and with a generalized linear mixed-effect model based on Poisson distribution for rearing and sniffing behavior. The CI resulted from 500 bootstrapping runs and 100 permutations.
