# Supplementary material for: Repeatability analysis improves the reliability of behavioral data
Source: PLoS One. 2020 Apr 2;15(4):e0230900. doi: 10.1371/journal.pone.0230900 (PMC7117744; doi:10.1371/journal.pone.0230900)
Supplement: S6 Table — Each repeatability value (R) was calculated over three adjacent days resulting in five groupings for average velocity and number of ambulations and in four groupings for rearing and sniffing behavior. For every factor R, the [2.5%, 97.5%] confidence intervals (CI) and p-values calculated by likelihood ratio test were displayed (n = 38 C57BL/6J, n = 15 BALB/cJ and n = 15 129S1/SvImJ male mice). Estimation of repeatability was conducted with a linear mixed-effect model based on Gaussian distribution for average velocity and ambulations and with a generalized linear mixed-effect model based on Poisson distribution for rearing and sniffing behavior. The CI resulted from 500 bootstrapping runs and 100 permutations. (PDF) [file pone.0230900.s010.pdf]

S6 Table. Repeatability values for strain as random factor for average velocity, ambulations as well was rearing and sniffing behavior.

	average velocity			ambulations			rearing			sniffing		
**grouping**	**R**	**CI**	**p**	**R**	**CI**	**p**	**R**	**CI**	**p**	**R**	**CI**	**p**
**day 1-3**	0.179	[0, 0.443]	1.2E-06	0.195	[0, 0.48]	7.57E-05	0.467	[0, 0.599]	3.93E-36	0.267	[0, 0.364]	NA
**day 2-4**	0.275	[0, 0.602]	3.58E-10	0.384	[0.009, 0.701]	1E-13	0.653	[0.009, 0.743]	6.63E-47	0.265	[0, 0.371]	NA
**day 3-5**	0.23	[0, 0.56]	3.51E-08	0.408	[0.005, 0.699]	3.95E-14	0.662	[0, 0.756]	5.74E-48	0.264	[0, 0.395]	NA
**day 4-6**	0.186	[0, 0.476]	1.3E-05	0.404	[0, 0.713]	5.15E-13	0.542	[0, 0.637]	8.13E-39	0.263	[0, 0.386]	NA
**day 5-7**	0.194	[0, 0.517]	1.62E-05	0.447	[0.013, 0.73]	1.68E-14	/	/	/	/	/	/

NA: close to zero or negative

Each repeatability value (R) was calculated over three adjacent days resulting in five groupings for average velocity and number of ambulations and in four groupings for rearing and sniffing behavior. For every factor R, the [2.5 %, 97.5 %] confidence intervals (CI) and p-values calculated by likelihood ratio test were displayed (n = 38 C57BL/6J, n = 15 BALB/cJ and n = 15 129S1/SvImJ male mice). Estimation of repeatability was conducted with a linear mixed-effect model based on Gaussian distribution for average velocity and ambulations and with a generalized linear mixed-effect model based on Poisson distribution for rearing and sniffing behavior. The CI resulted from 500 bootstrapping runs and 100 permutations.
