# Supplementary material for: Repeatability analysis improves the reliability of behavioral data
Source: PLoS One. 2020 Apr 2;15(4):e0230900. doi: 10.1371/journal.pone.0230900 (PMC7117744; doi:10.1371/journal.pone.0230900)
Supplement: S1 Text — (PDF) [file pone.0230900.s011.pdf]

S1 Text. R-script for the repeatability analysis. In the following you find a brief description for the repeatability analysis using the software R. (#) symbolize a comment; (>) symbolize a command. Animal ID, strain or experiment were included as random factors, whereas distance travelled (cm) or average activity (%) served as fixed factors. To avoid complications with R, please save the data table in .csv format.

```
# Install the rptR package (https://cran.r-project.org/web/packages/rptR/vignettes/rptR.html)

> install.packages(“rptR”)

# If you have already installed the rptR package, just load the package

> library(rptR)

# Set the location were you have saved your data table

> setwd(“server/name_of_the_folder”)

# load the data table

> daten=read.csv(“server/name_of_the_folder/name_of_data_table.csv”, sep=”;”, dec=”.”, header=TRUE)

# testing for normal distribution by Q-Q-norm plot

> qqnorm(daten$random_factor)

# Calculation of repeatability values including all random factors over the whole period. If the data is not normal distributed use ’Poisson’ distribution instead of ‘Gaussian’ distribution

> rep1 <- rpt(fixed_factor ~ (1| ID) + (1| strain) + (1| experiment), grname = c(“ID”, “strain”, “experiment”), data = daten, datatype= “Gaussian”, nboot=500, npermut=100)

# Observation of the progress of repeatability, repeatability values were calculated over three adjacent trails.

> daten =subset(daten, daten$trial==i |daten$trial==i+1 |daten$trial==i+2 )

> rep2 <- rpt(fixed_factor ~ (1 | random_factor), grname = c("random_factor"), data = daten, datatype = "Gaussian", nboot = 500, npermut = 100)

# Calculation of repeatability value of one random factor (e.g. animal ID) with adjustment for an identified effect covariate (e.g. strain)
```

```
> daten =subset(daten, daten$trial==i |daten$trial==i+1  |daten$trial==i+2 )

> rep3 <- rpt(fixed_factor ~ strain + (1 | ID), grname = c("ID"), data = daten, datatype = "Gaussian", nboot = 500, npermut = 100)
```
